# A medial prefrontal cortex-nucleus acumens corticotropin-releasing factor circuitry for neuropathic pain-increased susceptibility to opioid reward

**DOI:** 10.1038/s41398-018-0152-4

**Published:** 2018-05-21

**Authors:** Yuanzhong Kai, Yanhua Li, Tingting Sun, Weiwei Yin, Yu Mao, Jie Li, Wen Xie, Shi Chen, Likui Wang, Juan Li, Zhi Zhang, Wenjuan Tao

**Affiliations:** 1 Key Laboratory of Brain Function and Disease of Chinese Academy of Science, Department of Biophysics and Neurobiology, University of Science and Technology of China, Hefei, 230027 China; 2 Institute of Health Sciences and technology, School of Life Sciences, Anhui University, Hefei, Anhui 2300601 China; 3 Department of Anesthesiology and Department of Pain Management, The First Affiliated Hospital of Anhui Medical University, Hefei, Anhui 230022 China; 4 Department of Psychology, Anhui Mental Health Center, Hefei, Anhui 230022 China

## Abstract

Recent studies have shown that persistent pain facilitates the response to morphine reward. However, the circuit mechanism underlying this process remains ambiguous. In this study, using chronic constriction injury (CCI) of the sciatic nerve in mice, we found that persistent neuropathic pain reduced the minimum number of morphine conditioning sessions required to induce conditioned place preference (CPP) behavior. This dose of morphine had no effect on the pain threshold. In the medial prefrontal cortex (mPFC), which is involved in both pain and emotion processing, corticotropin-releasing factor (CRF) expressing neuronal activity was increased in CCI mice. Chemogenetic inhibition of mPFC CRF neurons reversed CCI-induced morphine CPP facilitation. Furthermore, the nucleus acumens (NAc) received mPFC CRF functional projections that exerted excitatory effects on NAc neurons. Optogenetic inhibition of mPCF neuronal terminals or local infusion of the CRF receptor 1 (CRFR1) antagonist in the NAc restored the effects of neuropathic pain on morphine-induced CPP behavior, but not in normal mice. On a molecular level, in CCI mice, CRFR1 protein expression was increased in the NAc by a histone dimethyltransferase G9a-mediated epigenetic mechanism. Local G9a knockdown increased the expression of CRFR1 and mimicked CCI-induced hypersensitivity to acquiring morphine CPP. Taken together, these findings demonstrate a previously unknown and specific mPFC CRF engagement of NAc neuronal circuits, the sensitization of which facilitates behavioral responses to morphine reward in neuropathic pain states via CRFR1s.

## Introduction

Non-medical abuse of prescription opioids has risen rapidly in recent years^1–4^, and how pain affects the likelihood of prescription opioid abuse has long been a topic of research and clinical interest^5–8^. However, few preclinical or clinical studies have addressed the interaction of pain and the rewarding effects of prescription opioids. To date, the neuroanatomical and molecular substrates underlying these processes remain poorly understood.

An important link between opioid reward and chronic pain is emotional processing, as drug reward induces a positive euphoric emotion, whereas pain is associated with a negative affective state^9–12^. Several brain regions, such as the amygdala, thalamus, medial prefrontal cortex (mPFC), and nucleus acumens (NAc), have been implicated in both chronic pain and emotional processing^11,13,14^. In particular, addictive substances can alter synaptic plasticity in both the mPFC and the NAc^15–18^. Meanwhile, NAc and mPFC neuronal activity is necessary for the full expression of neuropathic pain-like behavior^19,20^. Thus, connections involving the mPFC and NAc are most likely to be altered in chronic pain, leading to susceptibility to opioid reward^21^. However, the mechanisms of the cause and effect relationship between changes in neural circuitry and opioid reward have not yet been elucidated.

Corticotrophin-releasing factor (CRF), a 41-amino-acid peptide, was initially characterized as the primary neurohormone involved in controlling the body’s response to stress^22,23^. Later, it was found that CRF is widely expressed in the central nervous system and plays a major role not only as a stress hormone but also as a neuromodulator via the activation of the CRF type 1 receptors (CRFR1) or CRF type 2 receptors (CRFR2)^22,23^. Accumulating evidence has shown that the dysregulation of these brain CRF systems is heavily implicated in multiple psychiatric and mood disorders including drug use disorder^24–26^. For example, the activation of CRFR1 in the NAc induces a positive affective state^22^, and chronic CRFR1 blockage reduces heroin intake and dependence-induced hyperalgesia^27^. In addition, the antagonism of CRF1Rs or the reduction of CRF1R expression attenuates hyperalgesia associated with inflammatory, visceral, and neuropathic pain in animals^28–31^. These findings suggest that the CRF/CRFR system may bridge the mPFC-NAc functional circuit in chronic pain-promoted susceptibility of opioid reward. To test this hypothesis, in this study, we investigated the CRFergic mPFC-NAc circuitry and molecular mechanisms underlying opioid reward facilitation under chronic neuropathic pain conditions in mice.

## Materials and methods

### Animals

In all of the experiments, C57BL/6J, G9a^fl/fl^, *CRF-Cre* [strain B6(Cg)-Crhtm1(cre)Zjh/J], and *Ai9* [strain B6.CgGt(ROSA)26Sortm9(CAG-TdTomato)Hze/J] male mice (purchased from Charles River or Jackson Laboratories) at 8–10 weeks of age were used. Except during cannula surgery, the mice were housed five per cage in a colony with ad libitum access to water and food. They were maintained under a 12-h light/dark cycle (lights on from 0700 to 1900 hours) at a stable temperature (23–25 °C). All of the procedures were approved by the Care Committee of the University of Science and Technology of China.

### Animal model of neuropathic pain

Mice were deeply anesthetized with isoflurane before and during CCI of the sciatic nerve or sham surgery. A small incision was made in the left thigh to expose the sciatic nerve, and three consecutive loose chromic gut ligatures (4/0) about 1 mm thick were placed around the nerve. For the sham surgery, the nerve was isolated without nerve ligation. All efforts were made to minimize animal suffering, to reduce the number of animals used, and to utilize alternatives to in vivo techniques. Mice that did not show pain hyperalgesia after CCI surgery were excluded from further experiments. Mechanical sensitivity was tested with a single series of von Frey filaments on the glabrous surface of each hind paw in sequence every 5–10 min. The up-down method was used to assess pain threshold.

### Microinjection

We performed mPFC and NAc infusions as described previously. Before surgery, the mice were fixed in a stereotactic frame (RWD, Shenzhen, China) with a combination of xylazine (10 mg/kg) and ketamine (100 mg/kg) anesthesia. A heating pad was used to maintain mice’s core body temperature at 36 °C. For the local infusion of drugs, a 26-gauge double-guide cannula (Plastics One, Roanoke, VA, USA) was implanted and aimed at the mouse mPFC (anteroposterior, 1.7 mm from the Bregma; lateral, ± 0.25 from the midline; dorsoventral, −3.5 mm from the dura) or NAc (anteroposterior, 1.5 mm from the Bregma; lateral, ± 1 from the midline; dorsoventral, −7.0 mm from the dura). The implanted mice were housed individually and allowed to recover from the surgery for 5 d before the experiments. An internal stainless steel injector, which was inserted into the guide cannula (Plastics One, Roanoke, VA, U.S) and attached to 10-μl syringes (Hamilton) with an infusion pump (Micro4, WPI, USA) at a flow rate of 100 nl per min, was used for infusion of the vehicle (200 nl), CRF, NBI27914, or antisauvagine-30 into the nucleus. The injector was slowly withdrawn 2 min after the infusion. The behavioral assays were performed about 30 min after the infusion. Mice with missed injections were excluded.

For viral injection, 100–300 nl of virus (depending on its expression strength and viral titer) was injected at a rate of 30 nl/min using calibrated glass microelectrodes connected to an infusion pump. Cre-dependent virus rAAV-Ef1α-DIO-hChR2 (H134R)-mCherry-WPRE-pA (AAV-ChR2, AAV2/9, 1.63 × 10^13^ vg/ml, 200 nl) or rAAV-Ef1α-DIO-eNpHR3.0-EYFP-WPRE-pA (AAV-eNpHR3.0, AAV2/9, 1.18 × 10^13^ vg/ml) was delivered into the mPFC of *CRF-Cre* mice. 4 weeks later, the expression of mCherry was detected in the whole brain, and optogenetic manipulation was performed. rAAV-Ef1α- DIO-hM3D(Gq)-mCherry-WPRE-pA (AAV-hM3Dq, AV2/8, 2.69 × 10^13^ vg/ml) and rAAV-Ef1α-DIO- hM4D(Gi)-mCherry-WPRE-pA (AAV-hM4Di, AAV2/9, 3.69 × 10^13^ vg/ml) were used for chemogenetic manipulations 3 weeks after injection^32^. The viruses rAAV-Ef1α-DIO-mCherry-WPRE-pA (AAV2/8, 8.93 × 10^12^ vg/ml) and rAAV-DIO-EYFP-WPRE-pA (AAV2/9, 1.95 × 10^12^ vg/ml) were used as the controls. AAV-EF1a-mCherry-IRES-WGA-Cre (1 μl, Zhien, Hefei, China) was similarly infused. Unless otherwise stated, all of these viruses were packaged by BrainVTA (Wuhan, China). All of the mice were transcardially perfused with 0.9% saline followed by ice-cold phosphate buffer (0.1 M) containing 4% paraformaldehyde. Images of signal expressions were acquired with a confocal microscope (LSM 710, Zeiss, Germany). Animals with missed injections were excluded.

### Optogenetic manipulations in vivo

An optical fiber cannula was first implanted into the NAc of an anesthetized mouse in a stereotaxic apparatus. The implants were secured to the mouse skull with dental cements. Chronically implantable fibers (diameter, 200 μm; Newdoon, Hangzhou) were connected to a laser generator using optic fiber sleeves. The 5-min delivery of blue light (473 nm, 2–5 mW, 15 ms pulses, 20 Hz) or yellow light (594 nm, 5–8 mW, constant) was controlled by a Master-8 pulse stimulator (A.M.P.I., Jerusalem, Israel). The same stimulus protocol was applied to the control group of mice. Mice with missed fiber locations were excluded.

### Brain slice preparation

Acute brain slices were prepared, as described in previous studies^33^. Mice were deeply anesthetized with pentobarbital sodium (2%, w/v, i.p.) and intracardially perfused with ice-cold (4 °C) oxygenated modified NMDG artificial cerebrospinal fluid (NMDG ACSF) containing (in mM) 93 N-methyl-D-glucamine (NMDG), 2.5 KCl, 1.2 NaH_2_PO_4_, 30 NaHCO_3_, 20 HEPES, 25 glucose, 2 thiourea, 5 Na-ascorbate, 3 Na-pyruvate, 0.5 CaCl_2_, 10 MgSO_4_, and 3 glutathione (GSH) (pH: 7.3–7.4, osmolarity: 300–305 mOsm/kg). Coronal slices (300 mm) containing mPFC or NAcon were made using a vibrating microslicer (VT1200 S, Leica, Germany), and the slices were then incubated in a submersion-type holding chamber containing oxygenated ACSF at 35 °C throughout the experiments. Each brain slice was transferred into a slice chamber (Warner Instruments, USA) for electrophysiological recording and continuously perfused with standard perfusate at 2.5–3 ml/min at a temperature of 32 °C maintained by an in-line solution heater (TC-344B, Warner Instruments, USA).

### Whole-cell patch clamp recordings

Whole-cell patch clamp recordings were made from visually identified mPFC or NAc cells. Patch pipettes (3–5 MΩ) were pulled from borosilicate glass capillaries (VitalSense Scientific Instruments Co., Ltd., Wuhan, China) with an outer diameter of 1.5 mm on a four-stage horizontal puller (P-1000, Sutter Instruments, USA). The pipettes were filled with intracellular solution containing (in mM) 145 KsCl, 10 EGTA, 10 HEPES, 2 MgCl_2_, 2 CaCl_2_, and 2 Mg-ATP with osmolarity adjusted to 285–290 mOsm/kg and pH adjusted to 7.2 with KOH. In a subset of these experiments, CRF was added to the standard ACSF, and slices were incubated in this drug solution for at least 1 h before the experiments began. The current-evoked firing was recorded using a current-clamp mode (*I* = 0 pA). The threshold current for firing was defined as the minimum strength of current injection required to elicit at least one or two spikes. The signals were acquired via a MultiClamp 700B amplifier, low-pass filtered at 2.8 kHz, digitized at 10 kHz, and analyzed with Clampfit 10.7 software (Molecular Devices, Sunnyvale, CA, USA). If the series resistance changed by >20%, the experimental recording would be terminated immediately.

### Light-evoked responses

The optical stimulation was delivered using a laser (Shanghai Fiblaser Technology Co., Ltd., China) through an optical fiber 200 μm in diameter positioned 0.2 mm away from the surface of the brain slice. To test the functional characteristics of the AAV-ChR2, the fluorescently labeled neurons expressing ChR2 from the *CRF-Cre* mice after 3–4 weeks virus injection were visualized and stimulated with a blue (473 nm, 5–10 mV) laser light using a 5 Hz, 10 Hz, and 20 Hz stimulation protocol with a pulse width of 15 ms. To test the function of CRF fibers expressing ChR2 from the mPFC, a sustained photostimulation (473 nm, 3 s, 20 Hz, 15 ms) was delivered in the NAc.

### CPP

The general CPP procedure has been described in previous reports. In a two-chamber CPP apparatus (Huaibei Zhenhua Co., Ltd. China), the mouse was habituated and then conditioned for 30 min with saline or morphine in a single daily session of saline paired with one chamber in the morning and morphine paired with the other chamber in the afternoon for 3 or 5 days to induce CPP behavior. A CPP test (15 min) before the conditioning pre-test determined the baseline preference, and mice that spent >60% of the total time in the chamber (equipment bias) were excluded from the study for an unbiased CPP paradigm. CPP behaviors are presented as CPP scores, defined by the formula CPP Score=time in morphine-paired chamber−time in saline-paired chamber in seconds.

### Western blotting

NAc tissues were taken on day 21 after CCI surgery and were homogenized in 100 μl RIPA lysis buffer with fresh protease inhibitors. The lysates were centrifuged, and the supernatant was used for SDS-PAGE. The protein concentrations were determined using a Bio-Rad protein assay kit. The protein was mixed with SDS sample buffer, heated to 95 °C for 10 min, separated under reducing conditions on an 8 or 5% SDS-polyacrylamide gel, and transferred to a nitrocellulose membrane. Membranes were incubated in solution containing an antibody to CRFR1 (1:300, GTX130244, GeneTex), G9a (1:1000, Millipore), histone H3 dimethyl Lys9 (1:200, Active Motif), GAPDH (1:2000, Cell Signaling Technology), or β-tubulin (1:2000, Cell Signaling Technology) with agitation overnight at 4 °C. Membranes were incubated in secondary antibody to rabbit HRP (1:10000) or to mouse Ig HRP (1:20000) (Calbiochem) for 1 h at room temperature. The bands were detected using enhanced chemiluminescence (GE Healthcare Biosciences).

### Chromatin immunoprecipitation (ChIP) assays

NAc tissues were harvested and immediately cross-linked in 1% formaldehyde for 15–20 min. After washing, the NAc tissue was homogenized for 10–30 strokes in a cell lysis buffer. The homogenate was centrifuged, and the supernatant was removed. The extracted chromatin was sheared by sonication into 200–500 bp fragments and diluted 10-fold in ChIP dilution buffer. Ten percent of the pre-immunoprecipitated lysate was used as the “input” control for normalization. Samples were incubated with an antibody to G9a (Millipore) or H3K9me2 (Novus Biologicals). DNA and histones were dissociated with reverse buffer. Binding buffer was used for DNA precipitation and purification, and elution buffer was used to elute purified DNA from the columns. All of the buffers were provided in the ChIP kits.

### DNA quantification by real-time PCR

To quantify the level of G9a and H3K9me2 at the gene promoter of *CRFR1*, quantitative real-time PCR was conducted with SYBR Green Master kit (Applied Biosystems) to measure the amount of G9a- and H3K9me2-associated DNA, with adenine phosphoribosyltransferase (APRT, house-keeping mRNA) as the negative control. The following primers (Shenggong, Hefei, China) were used: *CRFR1* (5′-CCGCTGTCTCCACTTATCTT-3′ and 5′- TCCCTCGTTCGTTCACTCAT-3′); *APRT* (5′-TGCTGTTCAGGTGCGGTCAC-3′ and 5′-AGATCCCCGAGGCTGCCTAC-3′). Each PCR reaction was repeated at least three times independently. The analysis of relative quantification of templates was performed, as described previously. Signal difference was calculated by the formula ΔCt = (N_exp_−N_ave_) × Ct_ave_ (N_exp_, normalized Ct value of the target or Ct_target_/Ct_input_; N_ave_, mean N value for control; and Ct_ave_, mean Ct value for control).

### Immunohistochemistry

The mice were deeply anesthetized with pentobarbital sodium (50 mg/kg, i.p.) and sequentially perfused with saline and 4% (w/v) paraformaldehyde (PFA). The brains were subsequently removed and post-fixed in 4% PFA at 4 °C overnight. After cryoprotection of the brains with 30% (w/v) sucrose, coronal sections (40 µm) were cut on a cryostat (Leica CM1860) and used for immunofluorescence. The sections were incubated in 0.3% (v/v) Triton X-100 for 0.5 h, blocked with 10% donkey serum for 1 h at room temperature, and incubated with the CRF antibody, (1:1000, T-4037, Peninsula Laboratories), at 4 °C for 24 h, followed by the corresponding fluorophore-conjugated secondary antibodies (ThermoFisher) for 2 h at room temperature.

### Statistics and drugs

We conducted simple statistical comparisons using the paired Student’s *t*-test (two-tailed). Two-way ANOVA and Bonferroni’s post hoc analyses were used to statistically analyze the data from the experimental groups with multiple comparisons. All of the data are expressed as the mean ± SEM, and significance levels are indicated as *p *<* 0.05, **p *<* 0.01, and ***p *<* 0.001. OriginPro 2017 software (OriginLab Corporation, USA) and GraphPad Prism 5 (GraphPad Software, Inc., USA) were used for statistical analyses and graphing. Offline analysis of the data obtained from electrophysiological recordings was conducted using Clampfit software version 10.7 (Axon Instruments, Inc., USA). Drugs were purchased from Sigma-Aldrich (St. Louis, MO) or Tocris Bioscience (Ellisville, MO).

## Results

### Neuropathic pain facilitates morphine-induced preference behavior

**Fig. 1:**
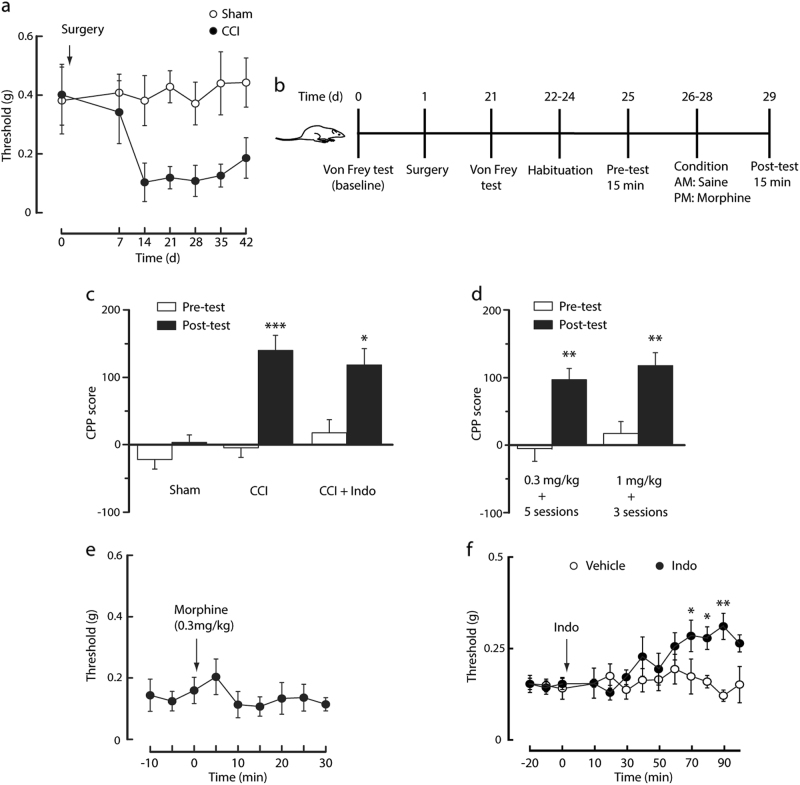
Persistent neuropathic pain facilitates morphine CPP behavior. **a** Time course of CCI-induced persistent sensory pain (F(1, 11) = 5.06, *p* = 0.0459, *n* = 6–7 mice). **b** Schematic of surgery and timeline of CCP experiments. **c** Preference behaviors before (pretest) and after 3 sessions of morphine conditioning (posttest, 0.3 mg/kg) in sham mice and mice treated with CCI or CCI plus indomethacin (Indo) injected 1 h before posttest (*n* = 8 mice per group). **d** Behavior of CPP in naive mice conditioned with different sessions and doses of morphine (*n* = 8 mice per group). **e** Effect of 0.3 mg/kg morphine on CCI-induced sensitization to mechanical pain (*n* = 5 mice per group). **f** Time course for changes in mechanical pain thresholds after CCI surgery, followed 21 days later by injection of vehicle or Indo (10 mg/kg, i.p.) at time 0 (F(1, 8) = 13.74, *p* = 0.0079, *n* = 5 mice per group). Data are expressed as mean ± *SEM*. *p *<* 0.01, **p *<* 0.01, ***p *<* 0.001

We used a well-accepted mouse model of neuropathic pain with a chronic constriction injury (CCI) of the sciatic nerve^34^. CCI-induced persistent mechanical pain sensitization, which lasted for at least 49 days after surgery (Fig. 1a). The morphine conditioned place preference (CPP) paradigm was used to determine the response to morphine reward, and we compared the rate of CPP induction by morphine conditioning sessions in CCI and sham mice from 21 days after surgery (Fig. 1b). In CCI mice conditioned daily with morphine at 0.3 mg/kg, a minimum of three conditioning sessions was required to induce significant CPP behavior; in contrast, in the sham mice, three such sessions were insufficient (Fig. 1c). Meanwhile, with five such sessions, or three sessions with 1 mg/kg morphine conditioning, CCP behavior was reliably established in the sham mice (Fig. 1d). Interestingly, 0.3 mg/kg morphine had no effect on CCI mice’s pain threshold 21 days after surgery (Fig. 1e). These results suggest that persistent neuropathic pain facilitates the acquisition of morphine CPP behavior, which is not due to morphine’s inhibition of pain. To confirm this hypothesis, indomethacin, a non-opioid analgesic agent, was used to inhibite the pain before each morphine conditioning (Fig. 1f). As expected, indomethacin pretreatment 1 h before each session of morphine paring did not significantly alter the facilitating effect of pain on CPP acquisition in CCI mice (Fig. 1c).

### Increased mPFC CRF neuronal activity contributes to morphine rewarding facilitation

**Fig. 2:**
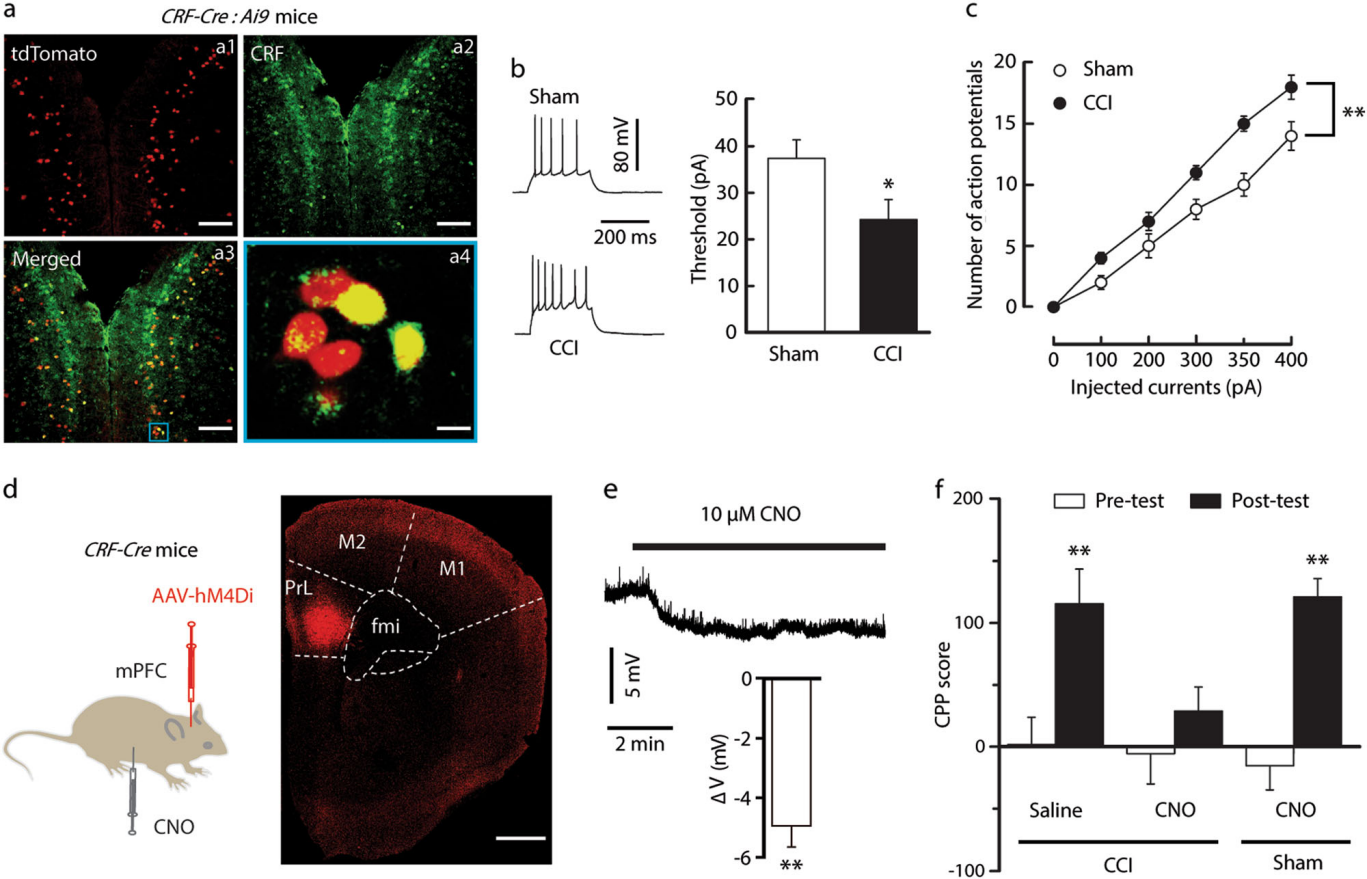
Neuropathic pain increases mPFC CRF neuronal activity, contributing to morphine reward facilitation. **a** Typical images of mPFC CRF neurons. CRF-Cre mice were crossed with Ai9 mice to produce transgenic mice with red tdTomato-expressing neurons (**a1**). The tdTomato signal was completely colocalized with the CRF (a2, green), based on immunofluorescence staining (a3 and a4, yellow). Scale bars=200 μm for a1, a2, and a3; 50 μm for a4. **b** and **c** The threshold (**b**) and number (**c**) of action potentials evoked by depolarizing current pulses of various amplitudes in CCI and sham mice (*n* = 15–22 neurons). **d** Schematic of mPFC infusion of Cre-dependent AAV-hM4Di and intraperitoneal (i.p.) injection of CNO in CRF-Cre mice (left). The right picture is the typical image of injection sites and viral expression within the mPFC. **e** A representative trace (top) from a whole-cell current-clamp electrophysiological recording showing that bath application of CNO hyperpolarized glutamatergic neurons in the mPFC and statistics (bottom) showing the average magnitude of hyperpolarization (*n* = 6 neurons). **f** Behavioral effects caused by mPFC injection of hM4Di-mCherry in CRF-Cre mice with CCI (0.3 mg/kg morphine) or sham (1 mg/kg morphine) surgery after i.p. injection of saline or CNO (*n* = 6–8 mice). The data are expressed as the mean ± SEM. *p *<* 0.05; **p *<* 0.01

Given the role of mPFC CRF signaling in drug addiction^24^, we wondered whether mPFC CRF neuronal plasticity participates in neuropathic pain-induced hypersensitivity to morphine reward. To visualize CRF neurons for whole-cell recordings in slices, *CRF-Cre* mice were crossed with *Ai9* (RCL-tdT) mice to produce transgenic mice with red tdTomato-expressing CRF (*CRF-tdT*). We found numerous *CRF- tdT* neurons in the mPFC (Fig. 2a). In response to a series of current injections, compared with sham *CRF-tdT* mice, we found a decrease in the threshold to elicit a spike in mFPC *CRF-tdT* neurons from CCI mice on day 21 after surgery and an increase in the spike number (Fig. 2b, c). Given the enhanced mPFC CRF neuronal activity in neuropathic pain, we then asked whether the inhibition of mPFC CRF neurons could restore the morphine response. Using Cre-inducible designer receptors that are exclusively inhibited by designer drugs (DREADD), system-Gi-protein-coupled receptor hM4Di and its ligand clozapine-*N*-oxide (CNO, 3 mg/kg)^35^, selectively to inhibit mPFC CRF neurons in *CRF-Cre* mice with CCI surgery (Fig. 2d, e), we found that the CPP behavior induced by three sessions of 0.3 mg/kg morphine conditioning was blocked (Fig. 2f). Interestingly, the morphine CPP behavior in sham mice was not affected by chemogenetic inhibition of mPFC CRF neurons (Fig. 2f). These results indicate that the increase in mPFC CRF neuronal activity contributes to morphine reward facilitation by persistent neuropathic pain.

### CRF mPFC → NAc pathway contributes to morphine reward in neuropathic pain

**Fig. 3:**
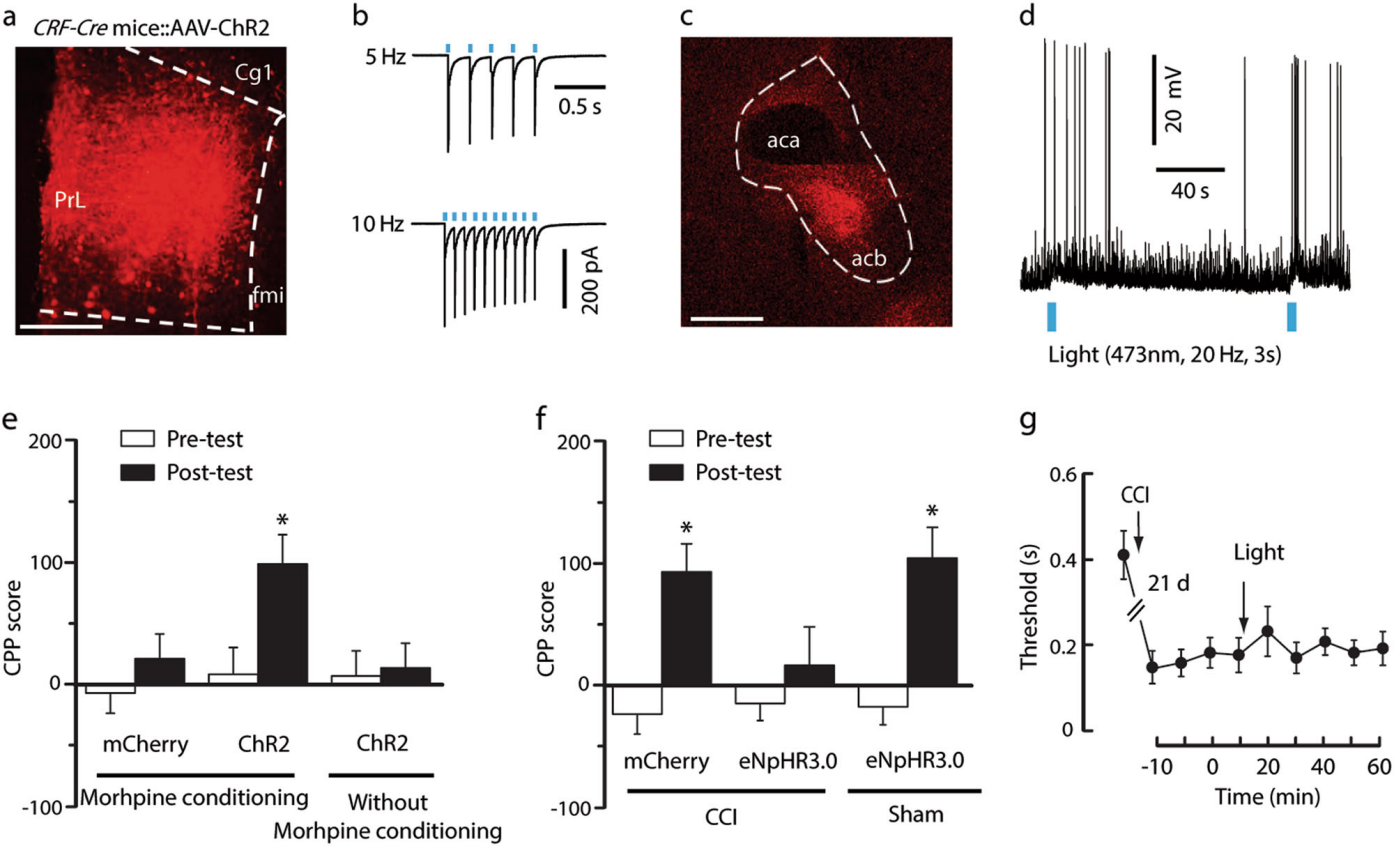
The CRF mPFC → NAc pathway contributes to the effects of neuropathic pain on morphine reward. **a** A typical image of AAV-ChR2-mCherry injection sites and viral expression within the mPFC in CRF-Cre mice. Scale bars=250 μm. **b** Sample traces of action potentials evoked by 473 nm light (blue bars) and recorded from AAV-ChR2-mCherry + neurons in acute brain slices. **c** Expression of mCherry in the NAc 3 weeks after mPFC infusion of AAV-ChR2-mCherry. Scale bars: 250 μm. **d** A sample trace of action potential firing of NAc neurons evoked by photostimulation (blue bars) in the NAc. **e** Behavioral effects of photostimulation in the NAc of CRF-Cre mice conditioned with or without 3 sessions of morphine (0.3 mg/kg) after mPFC infusion of AAV-ChR2 or control vector (*n* = 6–8 mice). **f** CPP behaviors in CRF-Cre mice with CCI (0.3 mg/kg morphine conditioning) or sham (1 mg/kg morphine conditioning) surgery after optical inhibition of mPFC CRF terminals in the NAc with mPFC infusion of AAV-eNpHR3.0 and photostimulation of the NAc (*n* = 5 mice per group). The control virus was AAV-mCherry. **g** Effects on CCI-induced sensitization mechanical pain in mice in (**e**) (*n* *=* 8 mice per group). Data are expressed as mean ± SEM. *p *<* 0.01

**Fig. 4:**
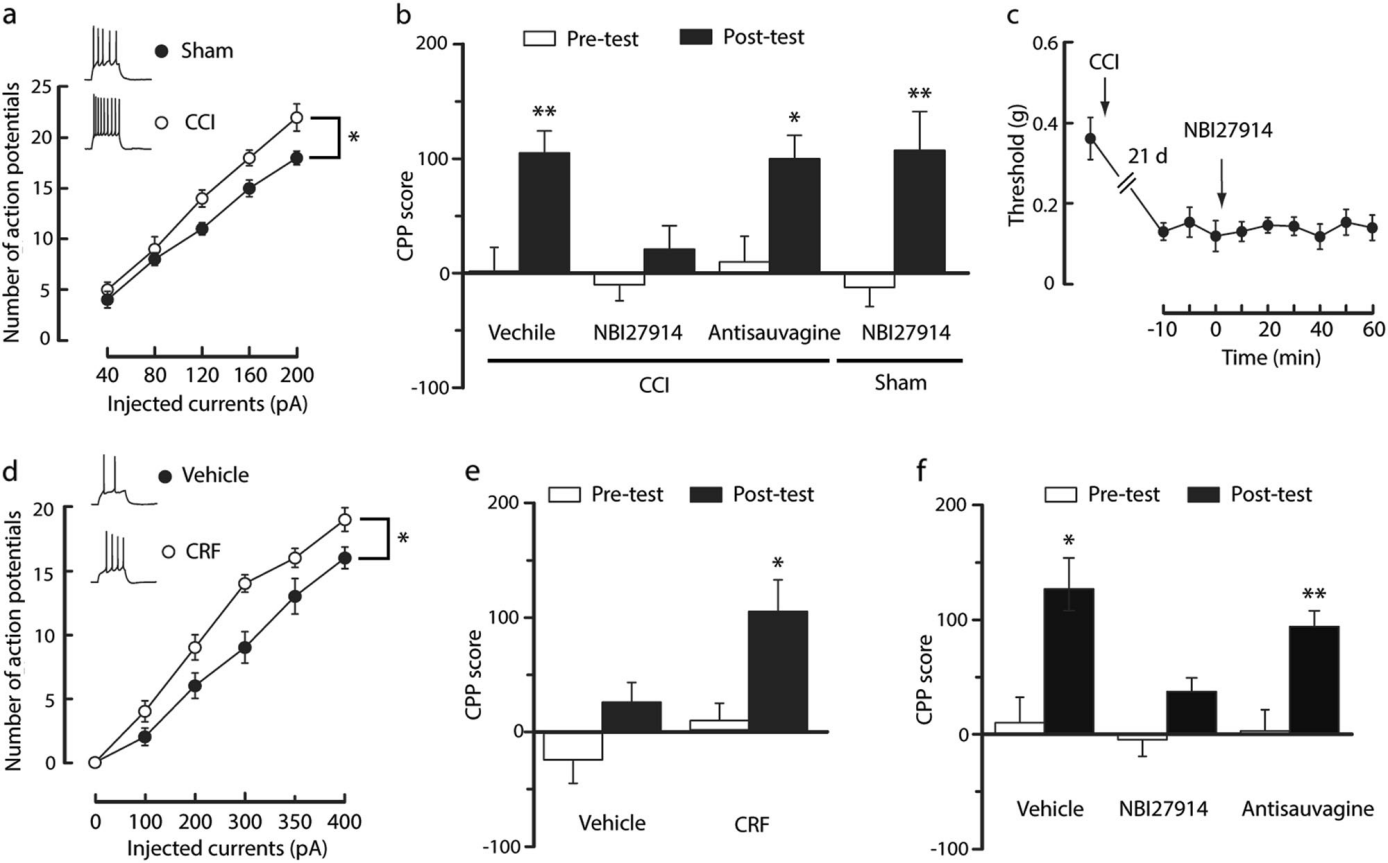
NAc CRFR1s in morphine reward hypersensitivity. **a** Evoked firing activity in NAc neurons from CCI and sham mice (*n* = 12–18 neurons). Insets are traces of action potentials from the indicated slice groups. **b** Morphine CPP behaviors in CCI (0.3 mg/kg) or sham (1 mg/kg) mice after mPFC infusion of vehicle, NBI27914, or antisauvagine-30 (*n* = 6 mice per group). **c** Effects of mPFC infusion of NBI27914 on pain threshold in CCI mice (*n* = 5 mice). **d** The action potential firing recorded in NAc neurons from sham mouse slices incubated in CRF or vehicle for at least 1 h (*n* = 10–16 neurons). **e** and **f** CPP behaviors in sham mice with the subthreshold dose of morphine conditioning with NAc infusion of vehicle, CRF (e), CRF plus NBI27914, or CRF plus antisauvagine-30 (f, F(2, 17) = 5.47, *p* = 0.0147) (*n* = 5–8 mice). Data are expressed as mean±SEM. *p *<* 0.01

We next identified the mPFC CRF output circuitry mediating the behavioral response in neuropathic pain. By mPFC infusion of Cre-dependent adeno-associated virus that expressed channelrhodopsin-2 (AAV-ChR2) in *CRF-Cre* mice, we observed mCherry^+^ (CRF) cell bodies in the mPFC (Fig. 3a), which displayed an excitatory response to light stimulation (Fig. 3b). Meanwhile, numerous mCherry^+^ fibers had emerged in the NAc (Fig. 3c). Whole-cell recording showed that optical stimulation of ChR2- containing mPFC CRF terminals in the NAc elicited action potential firing in brain slices (Fig. 3d). These results suggest that NAc neurons receive direct mPFC CRF projections, which exert excitatory effects. Behaviorally, optical activation of the mPFC CRF terminals in the NAc showed that CPP behavior was established by subthreshold morphine conditioning (0.3 mg/kg) in sham mice (Fig. 3e). In contrast, the morphine CCP behavior was blocked by optical inhibition of the mPFC CRF terminals in the NAc of CCI mice, but not sham mice (Fig. 3f), whereas the pain sensitization was not influenced (Fig. 3g). In addition, compared with the sham mice, the NAc neuronal firing rate was increased in the CCI mice (Fig. 4a). These results establish the functional relevance of the CRF mPFC → NAc pathway to morphine CCP behavior under neuropathic pain conditions, but not normal conditions.

An aversive stimulus produces effects that are sufficient to drive negative reinforcement, in which a modest positive reinforcer, e.g., a low dose of morphine (0.3 mg/kg), can drive rewarding effects^36,37^. To examine whether activation of this mPFC CRF pathway on its own produces a place aversion, we examined the behavioral consequences after activation of the mPFC → NAc pathway. The *CRF-Cre* sham mice with mPFC injection of AAV-ChR2 were conditioned for 30 min with or without blue light stimulation in the NAc in a single daily session, which is similar to pairing saline with one chamber in the morning and pairing morphine with the other chamber in the afternoon for 5 days. The results showed no preference or averse behaviors (Fig. 3e), which suggested that activation of the mPFC CRF pathway is not sufficient to drive negative reinforcement.

### NAc CRFR1s are involved in mPFC CRF actions on morphine reward in neuropathic pain

CRF acts on both CRFR1 and CRFR2. We determined which types of CRFRs were involved in CRF actions. With the infusion of CRFR1 antagonist NBI27914 (500 ng in 200 nl) or CRFR2 antagonist antisauvagine-30 (3.6 μg in 200 nl) into the NAc before each morphine conditioning session in CCI mice, we found that the CPP behavior was blocked only by NBI27914 and not by antisauvagine-30 (Fig. 4b), suggesting the specific role of CRFR1 under these conditions. Notably, NBI27914 had no effects on morphine CPP behavior in sham mice (Fig. 4b). In addition, this dose of NBI27914 had no effect on CCI-induced pain sensitization (Fig. 4c).

Next, we asked whether exogenous CRF could mimic the effect produced by CCI. After treatment of sham mouse slices with CRF (100 nM) for at least 1 h, NAc cells displayed increased action potential firing rates when compared with the cells in the vehicle-treated control slices (Fig. 4d). Furthermore, through the infusion of CRF (1.5 μg in 200 nl) into the NAc before each morphine conditioning session, CPP was established by three morphine conditioning sessions (0.3 mg/kg) in sham mice (Fig. 4e), which was inhibited by the co-infusion of NBI27914, but not by antisauvagine-30 (Fig. 4f). These results suggest that NAc CRFR1s, but not CRFR2s, are involved in mPFC CRF actions on neuropathic pain-increased susceptibility to acquiring CPP behavior.

### G9a-mediated *CRFR1* gene de-repression in neuropathic pain

**Fig. 5:**
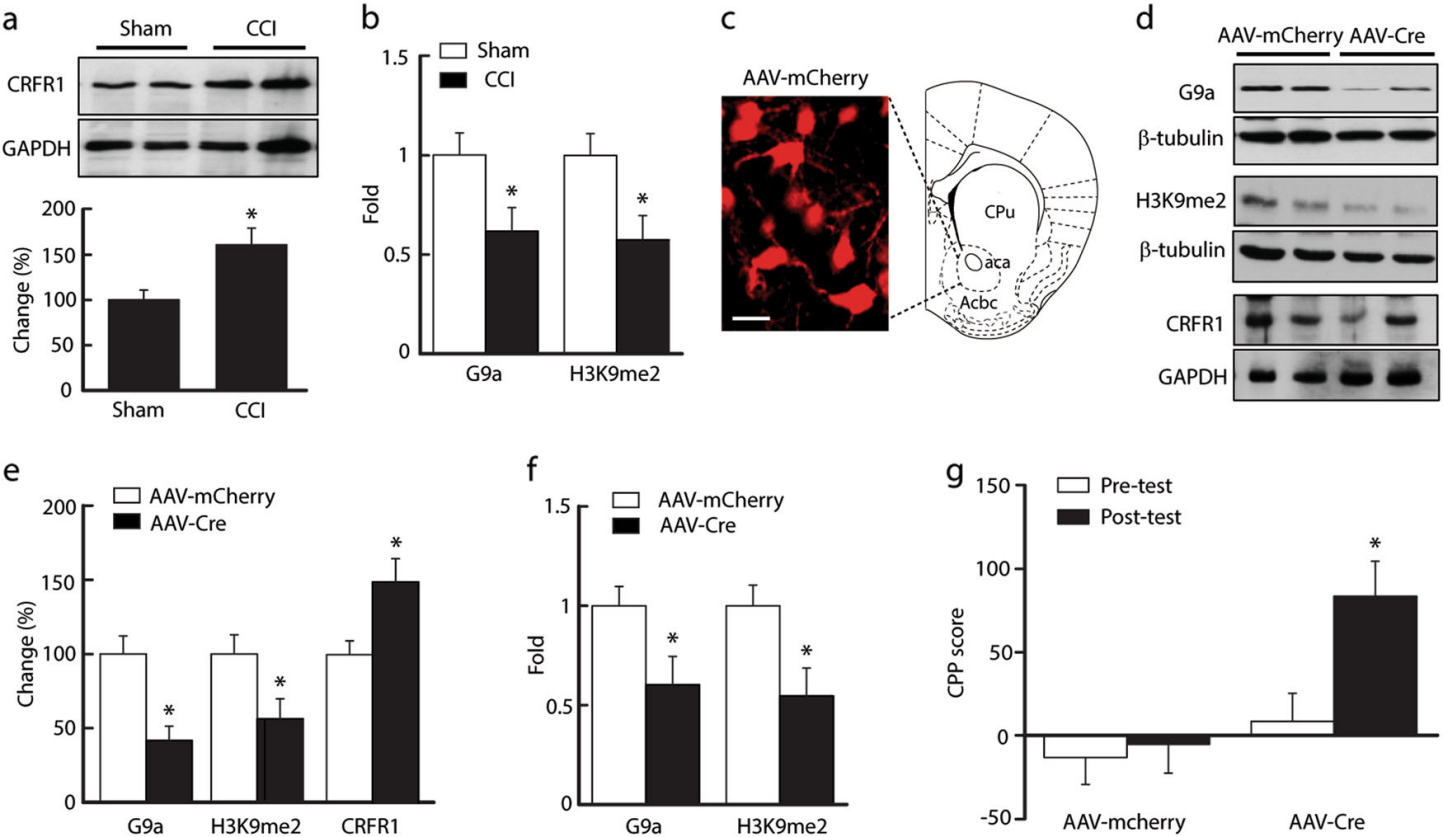
G9a represses CRFR1s. **a** Representative Western blots (top) and summarized results (bottom) of CRFR1 protein in the NAc from CCI and sham mice (*n* = 6 mice per group). The molecular mass was ~51 kD for CRFR1s and 37 kD for GAPDH. **b** Normalized levels of G9a and H3K9me2 across the CRFR1 promoter region in NAc tissues from mice in **a** (*n* = 8 mice per group). **c** Representative images of expression of an AAV-Cre vector infused in the NAc from a G9a^fl/fl^ mouse. Scale bars=250 mm. **d**, **e** Western blots (**d**) and summarized data (**e**) of NAc levels of G9a, H3K9me2, and CRFR1 in G9a^fl/fl^ mice after NAc infusion of AAV-GFP (control) or AAV-Cre (*n* = 6 mice per group). **f** Normalized levels of G9a and H3K9me2 across the CRFR1 promoter region in NAc tissues from mice in (**e**) (*n* = 6 mice per group). **g** CPP behaviors in naïve G9a^fl/fl^ mice conditioned with 0.3 mg/kg morphine after NAc infusion of AAV-GFP (control) or AAV-Cre (*n* = 8 mice per group)

Notably, *CRFR1* gene transcriptional activity is also altered in response to stress^38^. As shown in Fig. 5a, the amount of CRFR1 protein was increased by 61% in the NAc tissues taken from the CCI mice compared to that of the sham mice (*n* *=* 6 mice/group, *p* *<* 0.05).

Studies in rodents have shown that G9a specifically catalyzes the dimethylation of histone 3 at lysine 9 (H3K9me2), which is an epigenetic marker of transcriptional repression, and regulates *CRFR1* activity in both chronic pain and drug addiction^39–41^. We then determined whether G9a regulated the increased expression of CRFR1s. Chromatin immunoprecipitation (CHIP) assays showed that the level of G9a at the *CRFR1* gene promoter regions was reduced in the CCI mice (Fig. 5b; 61.8 ± 11.8% of the sham control, *n* *=* 9 mice per group, *p* *<* 0.05), and H3K9me2 changed accordingly along with G9a (Fig. 5b; 57.4 ± 12.2% of sham control, *n* *=* 9 mice per group, *p* *<* 0.05). These results suggest that CCI decreased CRFR1 expression via a G9a-mediated epigenetic mechanism.

To characterize the function of this epigenetic regulation of behavior response, we examined morphine CCP behavior after knockdown of G9a in the NAc using an AAVEF1a-mCherry-IRES-WGA- Cre (AAV-Cre) vector (Fig.5c). As shown in Fig. 5d and e, the infusion of AAV-Cre into the NAc of G9a^fl/fl^ mice significantly reduced the levels of G9a protein (41.7 ± 9.6% of the control, *n* *=* 6 mice per group, *p* *<* 0.05) and H3K9me2 (56.2 ± 13.7% of the control, *n* *=* 6 mice per group, *p* *<* 0.05), while the level of CRFR1 protein was increased (149.2 ± 15.6% of the sham control, *n* *=* 6 mice per group, *p* *<* 0.05). Meanwhile, G9a and H3K9me2 occupancy on the *CRFR1* promoters was reduced (Fig. 5f). Behaviorally, AAV-Cre-injected G9a^fl/fl^ mice displayed increased sensitivity to CPP behavior induced by a subthreshold dose of morphine when compared with the AAV-GFP-injected control mice (Fig. 5g).

## Discussion

Circuits comprising the mPFC are essential in processing emotion, and therefore are involved in the affective component of pain and drug reward^20,42–45^. A comprehensive understanding regarding the functionality of pain-relevant circuitry in the mPFC is lacking. We found that neuropathic pain increases the response to morphine reward, accompanied by increased mPFC CRF neuronal activity. Given that pain and cognitive processing are represented in overlapping regions, the mPFC circuitry may regulate the reciprocal relationship that exists between neuropathic pain and opioid reward. Previous studies have shown that GABAergic neurons in the central nucleus of the amygdala (CeA) are involved in pain facilitation of the reward response^46^, whereas the functional output of the CeA that produces these processes is unknown. It has been shown that pain-related hyperactivity in the amygdala leads to the deactivation of the mPFC and decision-making deficits, suggesting that functional interactions between the amygdala and PFC are important for emotional learning and behavior^45,47^. Thus, it is possible that the altered mPFC activity is triggered by pain-modulatory regions, such as the amygdala^48,49^, to produce morphine-reward facilitation. Of particular interest is that persistent inflammation does not change the amount of self-administered morphine in rats, but increases the morphine-seeking behavior after morphine withdrawal^50^. Both of these factors work to implicate the long-lasting effect of pain on the risk of opioid misuse. The subtle difference may result from differences in the species (mice vs. rats) and the behavioral paradigms (CPP vs. self-administration).

Increased functional connectivity between the NAc and the prefrontal cortex, which takes place through cortico-striatal-pallidal-thalamic loops, is predictive of the transition from acute to chronic pain^21,51,52^, indicating the clinical significance of this pathway. Our finding that mPFC CRF neurons project to the NAc to exert excitatory effects supports this notion. Interestingly, the inhibition of the CRF mPFC→NAc pathway blocked neuropathic pain effects on morphine reward, whereas it has no effect on pain sensitization. In fact, until recently, NAc was thought to encode salience for pain, while NAc valuation of acute analgesia was distorted in chronic pain patients^53^. Our results suggest that, at least for the CRF system, the mPFC → NAc pathway is involved in morphine susceptibility, but not pain relief, under neuropathic pain conditions. In addition, a previous study reported that when pain was acutely inhibited by analgesic agents, the acquisition of morphine CPP was still facilitated in inflammatory pain animals^46,54^. Consistent with this finding, when we used a non-opioid analgesic agent to inhibit pain before each morphine conditioning, the CPP behavior was not affected in CCI mice. Furthermore, our results showed that 0.3 mg/kg morphine, which induced CCP in CCI mice, had no effect on CCI-induced pain sensitization. These results indicate that the susceptible CPP response is due more to a heightened rewarding effect of morphine than to morphine’s inhibition of acute pain. Notably, in normal mice, inhibition of the mPFC → NAc pathway had no effects on morphine CPP behavior, whereas activation of this pathway facilitated CPP acquisition. These results suggest that persistent pain- induced CPP occurs through a specific mechanism.

It is presumed that pain is an inherent stressor^55^. CRF is thought to be a key molecular link between the behavioral effects of stress and drugs of abuse. Although the hypothalamic–pituitary–adrenal (HPA) axis, a source of CRF, plays a fundamental role in regulating the behavioral and neuroendocrine responses to stressors, whereas a previous study found CCI did not affect indices of basal HPA axis activity^56^. This suggests that the role of CRF in pain modulation may take place beyond the HPA. Although the CRF antibody is not ideal for CRF immunoreactivity in different species, including mice, rats and primates, we found that the mPFC is a CRF-rich brain region in mice. Persistent neural injury enhanced mPFC CRF neuronal activity, and chemogenetic inhibition of these neurons reversed CCI- induced morphine CPP facilitation. These results indicate that mPFC CRF is a critical modulator linking neuropathic pain and morphine reward hypersensitivity, which could extend beyond the glutamatergic and GABAergic systems in the mPFC. This notion is supported by the finding that the functional interactions between dopamine and CRF systems regulate drug abuse independently of the effects on the pituitary systems^27,30^. In addition, the amygdala CRF contributes to pain chronification and mediates the affective component of pain^31,57^. Whether the amygdala CRF is involved in persistent pain-increased morphine susceptibility needs further investigated.

Both the CRF1 and CRF2 receptors are involved in generating emotional pain responses^29^. The current study shows that mPFC CRF-mediated morphine reward hypersensitivity under neuropathic pain conditions develops through NAc CRFR1s, but not CRFR2s, which is consistent with previous findings that the activation of CRFR1 in the NAc induces a positive affective state^2^. In addition, the optical inhibition of mPFC CRF terminals in the NAc reversed the morphine response to neuropathic pain, but not pain sensitization, confirming that the CRF-CRF1 system in the mPFC → NAc circuit is not involved in the etiology and maintenance of CCI-induced allodynia. Of note, CRF/CRFR1 signaling has a complicated physiological effect. For example, CRF has both analgesic and anti-hyperalgesic effects in rodent pain models^28^.

Numerous types of epigenetic modifications within the brain’s reward circuitry in animal models of chronic pain and drug addiction have been investigated^58,59^. For example, chronic morphine downregulates G9a in the NAc, and G9a overexpression promotes analgesic tolerance and withdrawal^39,40^. The present study provides original evidence that G9a, through transcriptional de-repression of CRFR1 via deceased binding of H3K9me2 at the *CRFR1* gene promoter, promotes CCP behavior. This finding is in general agreement with the previous finding that the downregulation of G9a in the CeA by persistent inflammatory pain contributes to the preference behavior of morphine reward^54^. Furthermore, stress reduces the level of H3K9 trimethylation at the *CRFR1* gene promoter^38^. Thus, neuropathic pain induces the dual enhancement of CRFR1 expression and mPFC CRF inputs in the NAc in a manner that is persistent and reliable enough to maintain mPFC-NAc circuitry maladaptation, generating susceptibility to morphine reward. Although the levels of CRFR1 do not govern the dominant actions of CRF, our results suggest that CRFR1 is a potential cofactor to the physiological effects of CRF.

Collectively, the present study defines a specific mPFC → NAc CRF neuronal circuit through which persistent neuropathic pain increases the response to morphine reward. Central to this process is a circuit mechanism that involves the increased activity in NAc-projecting mPFC CRF neurons after chronic nerve injury. Meanwhile, the increased mPFC CRF input exerts excitatory effects on the NAc via CRFR1s, which is upregulated by a G9a-H3K9me2-mediated epigenetic mechanism. This convergence of regulating mechanisms provides a circuitry and molecular basis for understanding neuropathic pain-facilitated opioid reward effects.
